# Identification, Decomposition and Segmentation of Impulsive Vibration Signals with Deterministic Components—A Sieving Screen Case Study

**DOI:** 10.3390/s20195648

**Published:** 2020-10-02

**Authors:** Karolina Gąsior, Hanna Urbańska, Aleksandra Grzesiek, Radosław Zimroz, Agnieszka Wyłomańska

**Affiliations:** 1 Faculty of Pure and Applied Mathematics, Hugo Steinhaus Center, Wrocław University of Science and Technology, Wyspiańskiego 27, 50-370 Wrocław, Poland; 242966@student.pwr.edu.pl (K.G.); 243070@student.pwr.edu.pl (H.U.); agnieszka.wylomanska@pwr.edu.pl (A.W.); 2 Faculty of Geoengineering, Mining and Geology, Wroclaw University of Science and Technology, Na Grobli 15, 50-421 Wroclaw, Poland; radoslaw.zimroz@pwr.edu.pl

**Keywords:** mixture of signals, source separation, shock description, sieving screen, raw materials industry

## Abstract

Condition monitoring is a well-established field of research; however, for industrial applications, one may find some challenges. They are mostly related to complex design, a specific process performed by the machine, time-varying load/speed conditions, and the presence of non-Gaussian noise. A procedure for vibration analysis from the sieving screen used in the raw material industry is proposed in the paper. It is more for pre-processing than the damage detection procedure. The idea presented here is related to identification and extraction of two main types of components: (i) deterministic (D)—related to the unbalanced shaft(s) and (ii) high amplitude, impulsive component randomly (R) appeared in the vibration due to pieces of ore falling down of moving along the deck. If we could identify these components, then we will be able to perform classical diagnostic procedures for local damage detection in rolling element bearing. As deterministic component may be AM/FM modulated and each impulse may appear with different amplitude and damping, there is a need for an automatic procedure. We propose a method for signal processing that covers two main steps: (a) related to R/D decomposition and including signal segmentation to neglect AM/FM modulations, iterative sine wave fitting using the least square method (for each segment), signal filtering technique by subtraction fitted sine from the raw signal, the definition of the criterion to stop iteration by residuals analysis, (b) impulse segmentation and description (beginning, end, max amplitude) that contains: detection of the number of impulses in a decomposed random part of the raw signal, detection of the max value of each impulse, statistical analysis (probability density function) of max value to find regime-switching), modeling of the envelope of each impulse for samples that protrude from the signal, extrapolation (forecasting) envelope shape for samples hidden in the signal. The procedure is explained using simulated and real data. Each step is very easy to implement and interpret thus the method may be used in practice in a commercial system.

## 1. Introduction

A vibration signal measured on machine housing is usually a non-stationary one, with a complex, time-varying frequency structure. It may consist of deterministic, random, and transients (impulsive) components. All these components can be modeled, analyzed, and might be a source of important information about machine condition or process performance; however, all these components should be treated using different tools. Therefore, understanding of the signal’s nature and extraction of each component for further analysis is a critical pre-processing step in the condition monitoring.

In engineering applications, the condition monitoring is related to the tracking of amplitudes and frequencies of deterministic components related to shafts, meshes, etc. In such a case, any random, transient components are considered to be unwanted disturbances [1,2,3,4,5].

There is another serious class of solutions that plays with random signals assuming that local damage in gearboxes or bearings “produces” random signals with two important properties: impulsiveness and periodicity [6,7]. From that perspective, cyclostationary analysis is probably the most important solution [6,7,8,9].

As mentioned in [6], before cyclostationary analysis—an appropriate tool for random processes—the deterministic components should be removed. It is the so-called Wold decomposition [10]. In fact, this kind of signal decomposition inspired us to prepare this paper.

Our machine, a sieving screen, generates vibrations with three main components: deterministic—related to the unbalanced shaft to excite the upper deck to classify raw materials; random, impulsive, and non-cyclic component related to falling down or moving along the deck the oversized pieces of raw materials and—in the case of local damage in rolling element bearings—an impulsive, cyclic, weak random signal. The mixture of these components definitely requires separation for further analysis. The ultimate goal in condition monitoring of the sieving screen is related to damage detection in bearings. Bearings used in the sieving screen are subjected to overloading related to mentioned falling down pieces of ore as well as intentionally introduced unbalance of the shaft. Therefore, in fact, we have two mentioned phenomena: varying load (due to varying amount of ore to classify and unbalance of the shaft) and impulsive load considered to be impulsive background noise. As discussed in research works published already [11,12], for this class of machines the problem of local damage detection is challenging and may be defined as the detection of cyclic impulsive signal in presence of non-cyclic impulsive noise [12,13,14,15,16,17,18,19].

The presence of non-Gaussian noise plays a crucial role here. If the level of impulsive disturbances is relatively small, one may use classical diagnostic techniques. When impulsive, non-Gaussian noise becomes a dominating component (the amplitude of a single impact is higher 20–30 times than cyclic impulses), most of the classical techniques used for diagnostics are non-effective. In the considered case, the situation is very dynamic. If the mined material delivered to the screen is well fragmented (depends on geology, blasting procedure, etc.), it may not contain the oversized pieces, so high amplitude impulses will not be present. However, if the oversized pieces of material are present in the material stream, a high level of disturbance will complicate diagnostic procedures. Thus, the key issue is to recognize if we have unwanted, non-cyclic impulses in the signal.

The motivation of this paper is to identify these non-cyclic impulses with high amplitude and to use information about their presence and properties in decision making related to damage detection. In other words, we would like to detect high energy impulses in the raw signal, segment them, and remove them from the diagnosis process. To detect high amplitude impulses, to estimate their duration, and to remove such a part of the signal may be seen as a very easy task. Unfortunately, due to a specific use case, a simple problem becomes more complicated and requires a special procedure.

A raw signal contains high energy deterministic components, so decomposition into random and deterministic signals should be performed first. Without this step, the precise localization of the impulse may be difficult.

Due to the complexity of the mining process, the input materials stream is time-varying (volume/mass is changing in time). It leads to a time-varying load for the sieving screen drive unit and, in consequence, it will bring slight frequency modulation for deterministic components (rotational speed will be time-varying). From a signal processing point of view, it is frequency modulation when a modulating signal is both deterministic (sine function due to shaft unbalance) and random process (material volume variation).

A decomposed random signal is then processed for impulse properties estimation. We need information related to the beginning, amplitude, and end of the impulse. Finding the maximum value of the impulse is in our case easy. However, precise detection of the beginning and especially the end of impulse may be challenging as they may be still hidden in background noise. Thus, we propose to identify impulse behavior using statistical analysis, envelope analysis, and simple curve approximation (fitting).

As was mentioned, in our case there are several well-known tasks in signal processing, namely, signal decomposition, modelling, detection, and segmentation. They are fundamental in signal processing so one may find many references related to them.

Signal decomposition in our context means decomposition into random and deterministic components (the Wold Decomposition). As we know, the source of determinism in the signal (unbalance of the shaft) can be approximated by a sine-wave function. When the deterministic signal is estimated, then the residual signal could be obtained by simple subtraction of the deterministic component from the raw signal. It is a very popular technique used in many applications, see [10,20,21,22]. In the case of several sine components, it could be done in an iterative way or the model could contain several sine components (we have proposed criteria for such iteration). It is worthy to mention that due to slight frequency modulation we use sine function fitting for a short segment of the signal (it does not work for the whole signal). It should be also highlighted that we “model” deterministic components to remove it from the signal, we do not pay attention to them later.

Obviously, the problem of deterministic part extraction is well described in the literature and many advanced techniques could be found as time-synchronous averaging, adaptive filters based on linear prediction theory, Vold-Kalman filters, etc. Even more papers are focused on non-stationary signal modelling [23,24,25,26,27,28]. Mostly they considered time-frequency domain (STFT, PWVD, wavelets, chirplets, etc.) [29,30,31,32]. A special case of the non-stationary signal is AM/FM modulation (this is also our case) exists in the vibration signal due to changing operating conditions or existing damage [33,34,35]. However, as our signal is not very complicated (at least the deterministic part), we decided to use the simplest solution for modelling of this component.

After deterministic components removal, in theory the purely random signal appears. In our case, it consists of the impulsive disturbances related to falling rocks and noise. Our next step is to identify each impulse in an automatic way taking into consideration the specific nature of the impulses. Impulse detection is a classical task in signal processing. In condition monitoring, it is related mostly to local damage detection and a variety of detectors can be used. In our context, the problem is simplified as the amplitude of the impulse is high and it is clearly visible in the raw signal, so simple statistical detectors can be used (peak, kurtosis, etc.) [36].

However, we would like to get precise information about impulses (when appeared, how strong it is, when disappeared in background noise). For such a task one may use signal segmentation. The signal segmentation is considered for random signals (this is a reason we need deterministic/random components separation). The segmentation is usually applied for a signal with essential changes of its properties (changes of the regime) [37,38] or extraction a specific pattern [39]. It is commonly used in many applications (biomedical signals, experimental physics, speech analysis, econometrics, seismic signal analysis, machine performance analysis, aircraft noise, physical sciences, etc.) [40,41,42,43,44,45,46].

In our case, we propose a procedure for segmentation that is based on hybrid statistical modelling. First, we detect the beginning of impulse using analysis of detected maximum values in decomposed signal, second, we exploit a concept of damped vibration and we fit the envelope (Hilbert-based) to the signal for samples that protrude from the signal. Knowing the shape of the envelope (exponential function), we can extrapolate it (forecast) for samples hidden in the signal.

The paper is organized as follows: in Section 2 we describe the machine, the experiment, and the data. In Section 3 we present the methodology used in the data processing which consists of four steps including iterative removal of deterministic components, determining the number of impulses, and identifying their location in the signal. In Section 4 we demonstrate the efficiency of the proposed methodology for simulated data. In Section 5 we analyze a real signal. The last section concludes the paper.

## 2. Experiment and Data Description

### 2.1. Machine Description

**Figure 1:**
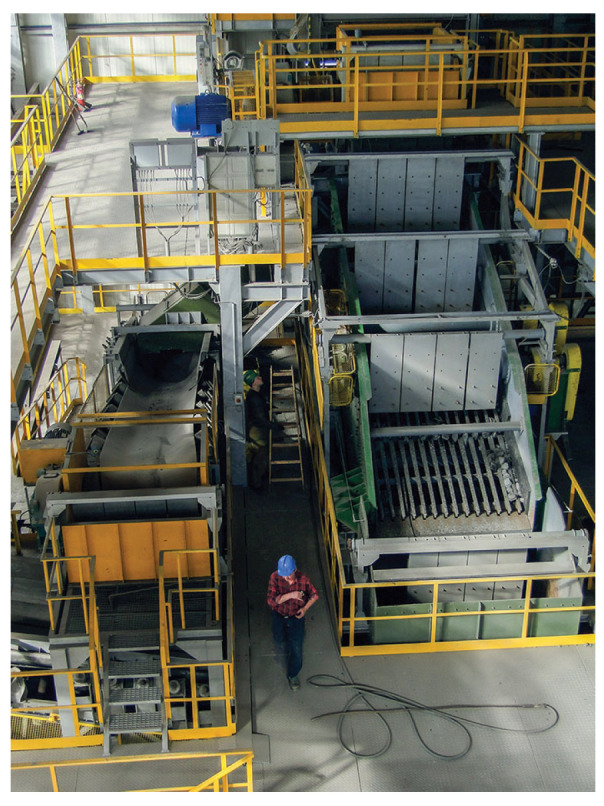
Vibrating screen machine (source: https://kghm.com/pl/w-pracy).

**Figure 2:**
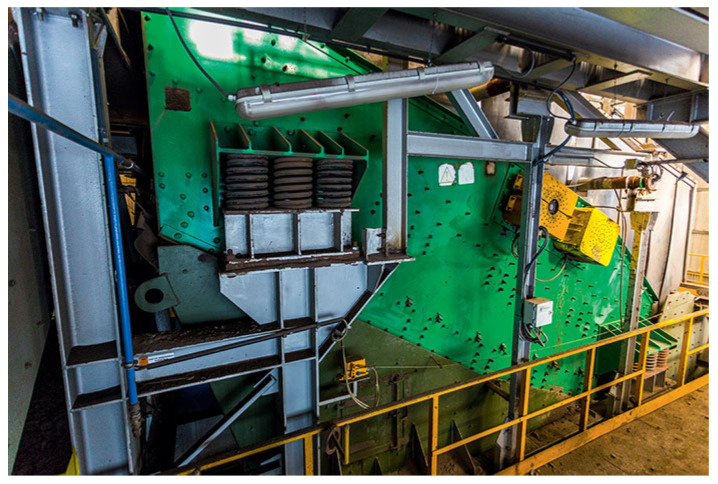
Vibrating screen machine (source: https://kghm.com/pl/w-pracy).

In the considered machine (see Figure 1 and Figure 2), a sieving screen is designed to separate the input raw material into three classes: less than 40, 40–110, and more than 110 mm. The material stream is coming into the screen (falling on the deck) from a conveyor located on the top. The screen supports consist of four sections with three springs in each corner. The screen is driven by two electric motors and individual belt transmissions. Special spherical roller bearings are used on the shafts of unbalanced exciters, see [12] for more details.

### 2.2. Experiment Description

An experiment was a passive one, we have measured vibration during normal operation of the machine with various types and quality of input material. As the screen is a critical component in the production line, no modification or stoppage was possible. During the experiment, many times the oversized pieces of ore hitting the deck have been noticed. It is a normal situation as blasting technology used in the mine provides various sizes of ore pieces. We have used typical devices for vibration measurements.

### 2.3. Data Description

During the experiment, many vibration signals from several channels have been recorded. Sampling frequency was set to 50 kHz to assure a wide range of frequency for transient impulsive data. The duration of the signal was c.a. 2 min to avoid too big to handle data files with multi-channel signals. Due to NDA, we are not allowed to provide more detailed information.

## 3. Methodology

In this section, we present the methodology used in real data analysis. The main goal of this procedure is to identify the location of the impulses in the acoustic signal presented in Section 2. The proposed methodology can be divided into four stages:

- iterative removal of deterministic components from the raw data,
- determining the number of impulses in a cleansed signal,
- detection of the moments when the impulses begin,
- detection of the moments when the impulses end.

**Figure 3:**
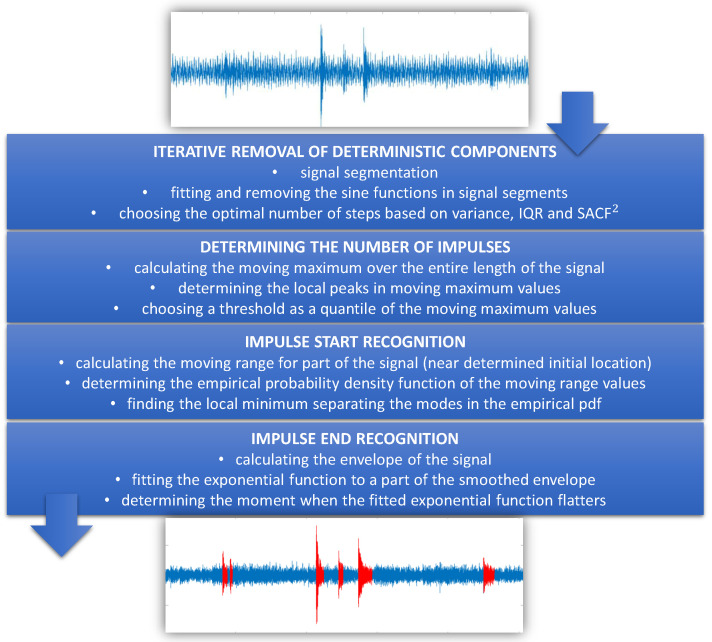
A scheme presenting the overall procedure. More details are provided in the Section 3.1, Section 3.2, Section 3.3 amd Section 3.4.

A scheme illustrating the overall procedure is presented in Figure 3 and the methods used in the subsequent stages are described in detail in the following subsections. Moreover, the performance of the proposed procedure is verified in Section 4, where a simulated signal is analyzed.

### 3.1. Iterative Removal of Deterministic Components

**Figure 4:**
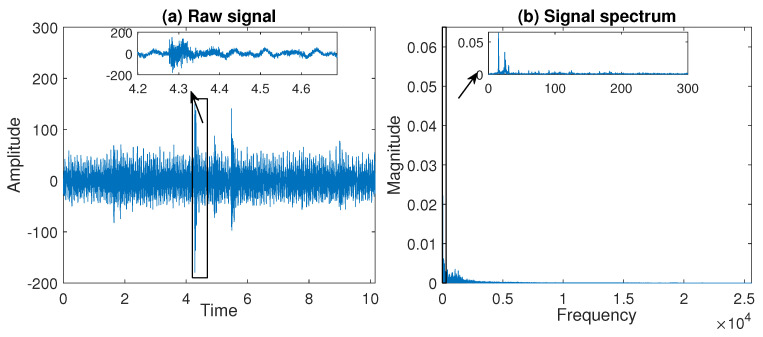
The trajectory of a raw signal—panel (**a**), and the signal spectrum—panel (**b**) for the real data analyzed in Section 5.

On the raw data plot in Figure 4a and in the signal spectrum in Figure 4b one can recognize the periodic trends that are present along the entire length of the signal. However, the frequencies of the periodic functions observed in the data change in time because, as it was already mentioned in the Introduction, the material stream coming into the sieving screen affects the engine operation. Therefore, to clean the data from the deterministic components, first, we divide the signal into segments. To choose the number of segments, we propose to compare the frequencies of the sine functions fitted to each segment of the signal for all segment lengths changing from 10,000 to 55,000 with a step equal to 5000 (by the segment length we mean the number of samples in one segment). As the best length, we choose the one for which the sine frequencies in the subsequent segments have the smallest variance.

Now, let *m* and *n* indicate the number of segments in the signal and the length of one segment, respectively. Then, $x_{i}=\left(x_{i,1},x_{i,2},\ldots ,x_{i,n}\right)$ denotes the observations in the *i*-th segment where i=1,2,…,m. In the first step, we fit a sine function to each segment $x_{i}$, i=1,2,…,m separately, using the least square method, and subtract the fitted function from the raw data. Let $r_{i,1}$, i=1,2,…,m indicate the residual time series obtained after removing the first sine function from the data. Next, we repeat the above step three more times and finally compare the raw signal and the obtained residual time series by calculating some statistics of $x_{i}$, $r_{i,1}$, $r_{i,2}$, $r_{i,3}$, and $r_{i,4}$ for the subsequent segments i=1,2,…,m. More precisely, we compare the variances, the interquartile ranges and the sums of squared auto-correlation functions computed for all segments. Let us mention here that since for the observations $y=(y_{1},y_{2},\ldots ,y_{n})$ the empirical auto-correlation function at *k* takes the following form [10]

(1) $$\hat{\mathrm{ACF}}\left(k\right)=\frac{\hat{\gamma }\left(k\right)}{\hat{\gamma }\left(0\right)},$$

where

(2) $$\hat{\gamma }\left(k\right)=\frac{1}{n}\sum_{j=1}^{n-k}\left(y_{j+h}-\bar{y}\right)\left(y_{j}-\bar{y}\right),$$

for $k=0,1,\ldots ,k_{max}$ and $0<k_{max}\leq n$, the mentioned sum of squared auto-correlation function is defined as follows

(3) $${\mathrm{SACF}}^{2}=\sum_{k=0}^{k_{max}}{\left(\hat{\mathrm{ACF}}\left(k\right)\right)}^{2}.$$

Since the auto-correlation function quantifies the interdependence in the data, after removing the deterministic components the value taken by ${\mathrm{SACF}}^{2}$ given in Equation (3) should decrease in comparison to the statistic computed for raw signal because the strong dependence arising from the presence of deterministic components disappears. Therefore, based on the values taken by ${\mathrm{SACF}}^{2}$ and other mentioned statistics, we decide which of the cleaned time series, $r_{i,1}$, $r_{i,2}$, $r_{i,3}$, or $r_{i,4}$, should be taken for the analysis in the further stages.

### 3.2. Determining the Number of Impulses

To determine the number of impulses in the signal cleaned in the previous step, we calculate the moving maximum over a sliding window of length $l_{1}$. We start from the first $l_{1}$ observations and move one sample to the right until there are enough elements to fill the window. In the next step, we compute the local peaks of the values taken by the moving maximum. As a local peak, we consider a data sample that is larger than its two neighboring samples. Then, the number of impulses in the signal is equal to the number of local peaks that are located above or on the level of a threshold chosen based on the calculated moving maximum values. For both considered simulated and real signals, the window length is equal to $l_{1}=9000$ and the threshold value is chosen as a certain quantile of the values taken by the moving maximum (quantile of order 0.7 or 0.875 for simulated and real data, respectively).

### 3.3. Impulse Start Recognition

To locate where the impulse starts, we consider a part of the signal that contains samples both preceding and following the beginning of the impulse. For this part of the signal, we calculate the moving range over a sliding window of length $l_{2}$ similarly as in the previous stage of the analysis, see Section 3.2. Then, we examine the empirical probability density function (pdf) of the computed moving range values. If the considered part of the signal contains the beginning of the impulse, the distribution is bi-modal and the local minimum separating the modes can be treated as a threshold. Namely, the values taken by the moving range, which are greater than the set threshold, indicate the area of the impulse, and the first value meeting this condition determines the beginning of the impulse. We recall that to estimate the empirical probability density function from the observations $y=(y_{1},y_{2},\ldots ,y_{n})$ one can use the kernel density estimator of f(y) defined as follows [47,48]

(4) $$\hat{f}\left(y\right)=\frac{1}{nh}\sum_{i=1}^{n}K\left(\frac{y-y_{i}}{h}\right)\;\mathrm{for}\;y\in R,$$

where K(·) is the non-negative kernel smoothing function, and *h* denotes the bandwidth. By choosing the kernel smoothing function, we determine the shape of the curve that is used to estimate the empirical pdf. For our purpose, we apply the Gaussian kernel of the following form

(5) $$K\left(y\right)=\frac{1}{\sqrt{2\pi }}\exp\left\{-\frac{y^{2}}{2}\right\}\;\mathrm{for}\;y\in R.$$

The bandwidth *h* is chosen according to the Silverman’s rule of thumb to be optimal for estimating Gaussian densities, see [49]. The mentioned method is implemented in many mathematical packages (e.g., “ksdensity” in Matlab). Moreover, for both simulated and real signal, the window length for the moving range is chosen as $l_{2}=20$.

### 3.4. Impulse End Recognition

Since each impulse present in the signal is a damped vibration, its envelope can be approximated by an exponential function. As the end of the impulse, we can consider the moment when the fitted exponential function flatters. Let $r=(r_{1},\ldots ,r_{N})$ indicate the signal cleaned at the first stage of the analysis, see Section 3.1, where *N* denotes its length. To extract the envelope of r we use the Hilbert transform. Namely, let us consider the analytic signal composed as follows

(6) $$ℵ\left(r\right)=r+i\;\tilde{r},$$

where $\tilde{r}=\left(\tilde{r_{1}},\ldots ,\tilde{r_{N}}\right)$ is the Hilbert transform of the original data, see [50]. Then, the envelope of the signal is given as the absolute value of the analytic signal given in Equation (6), namely |ℵ(r)|. In the next step, we smooth the envelope of the signal using the simple two-sided moving average method, see [10], and we fit an exponential function to the smoothed envelope of the impulse staring from the peak found at the previous stage of the analysis, see Section 3.2. As the end of the approximation interval, we choose the point where the envelope intersects the threshold determined based on the standard variation of the signal. In addition, consequently, as the end of the impulse, we consider the point where the fitted exponential function is flattered. In practice, we identify the moment when the difference in values taken by the fitted function for adjacent samples takes a value less than the selected ϵ. For real signal, the value of ϵ is chosen to be equal to $5.52\times 10^{-4}$.

## 4. Simulated Data Analysis

**Figure 5:**
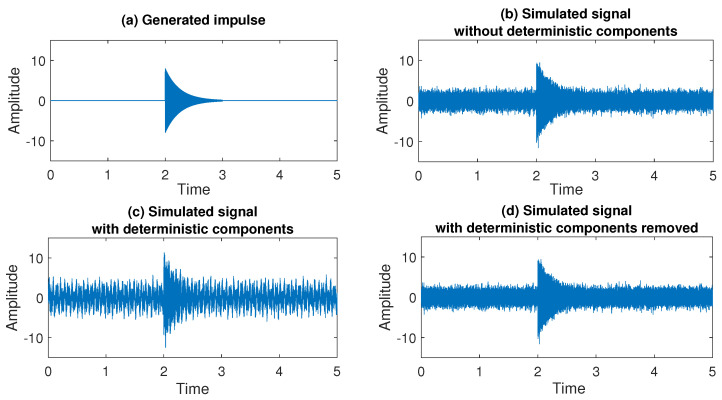
Generated impulse (**a**), simulated signal without and with deterministic components, (**b**) and (**c**) respectively, and simulated signal after iterative removal of two sine functions (**d**).

In this section, we apply the proposed methodology to the simulated data presented in Figure 5. The analyzed signal is generated as a sequence of independent observations drawn from the standard normal distribution. It is simulated on the interval [0,5] with sampling frequency equal to 10,000 Hz. Additionally, starting at t=2 we generate an exponentially decaying impulse of length 1000 that follows the formula

(7) f(t)=aexp(−bt)sin(2πft),

where a=25,000, b=4 and f=3000, see Figure 5a for the generated impulse and Figure 5b for the simulated signal. In the first subsection, we demonstrate the performance of the method related to the removal of deterministic components proposed in Section 3.1. For this purpose, to the simulated signal we add additional deterministic components, namely two sine functions (with different amplitudes and frequencies) of the form

(8) $$s_{i}\left(t\right)=A_{i}\sin\left(2\pi \;f_{i}\;t\right)$$

for i=1,2, where $A_{1}=1.4$, $A_{2}=1.2$, $f_{1}=15$, and $f_{2}=25$. The simulated signal with deterministic components is presented in Figure 5c. After verifying the performance of the method for removing determinism, in the subsequent subsections, we will focus on the remaining stages of the proposed procedure described in Section 3.2, Section 3.3 and Section 3.4, namely determining the number of impulses and their location in the signal.

### 4.1. Simulation Study for Iterative Removal of Deterministic Components

Since in the simulated signal analyzed here, the parameters of $s_{1}\left(t\right)$ and $s_{2}\left(t\right)$ do not change over time, we proceed by omitting the step of dividing the signal into segments. Then, according to the proposed procedure, we iteratively fit the sinusoidal functions and determine the values of variance, interquartile range, and ${\mathrm{SACF}}^{2}$ of the residual signals. Since we only have one segment in the signal, the statistic values after subtracting one, two, three, and four sine functions are presented in Table 1.

As one can notice, the statistics values change most significantly after subtracting the first and second sine function. The third and the fourth sine fitted to the data do not change the statistics values so significantly. Additionally, the sine functions fitted in the first two iterative steps have a form that is very similar to the functions added to the original signal, namely

(9) $${\tilde{s}}_{1}\left(t\right)=1.40\sin\left(30\pi \;t+0.02\right)\;\mathrm{and}\;{\tilde{s}}_{2}\left(t\right)=1.21\sin\left(50\pi \;t+0.01\right).$$

The signal reconstructed after subtracting two sine functions is presented in Figure 5d. We can see that visually it is very close to the one presented in Figure 5b. Additionally, the mean squared error of the original signal without deterministic components and the signal reconstructed by subtracting two sinusoidal functions is less than 0.0002 that confirms the efficiency of the method.

### 4.2. Simulation Study for Impulse Location Detection

**Figure 6:**
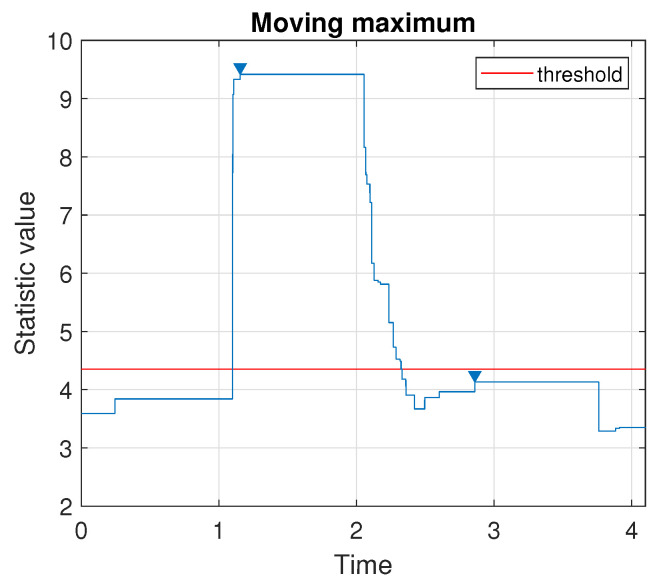
Values taken by the moving maximum calculated over a sliding window of length 9000. Triangle markers indicate the local peaks and the threshold is chosen as a quantile of order 0.7.

To find the location of the generated impulse, first, we apply the method determining the number of impulses in the signal. According to the procedure presented in Section 3.2, we plot the values taken by the moving maximum, see Figure 6. Since there is one local peak located above a chosen threshold (a quantile of order 0.7), we conclude that there is only one impulse in the signal.

**Figure 7:**
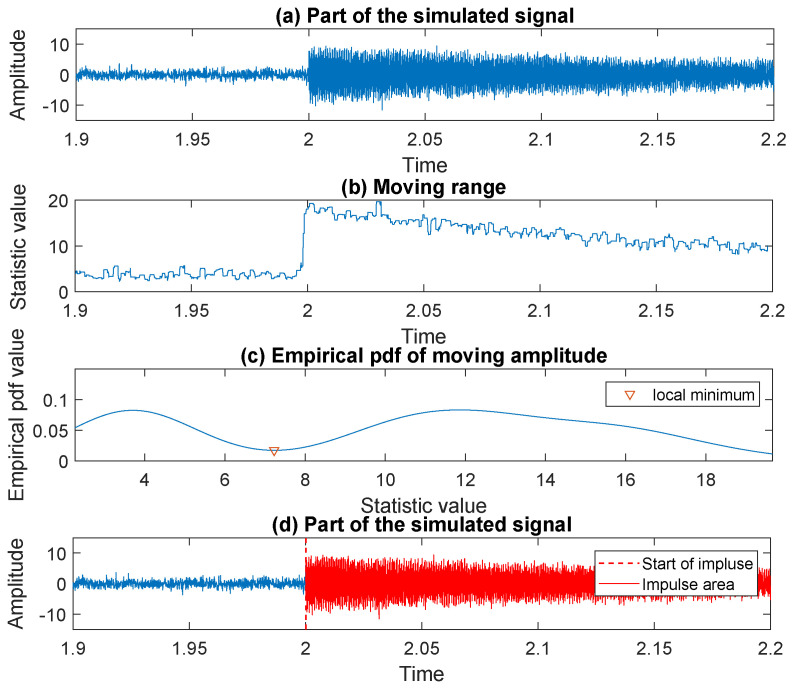
The consecutive steps of the procedure leading to the start recognition for an impulse in the simulated signal.

**Figure 8:**
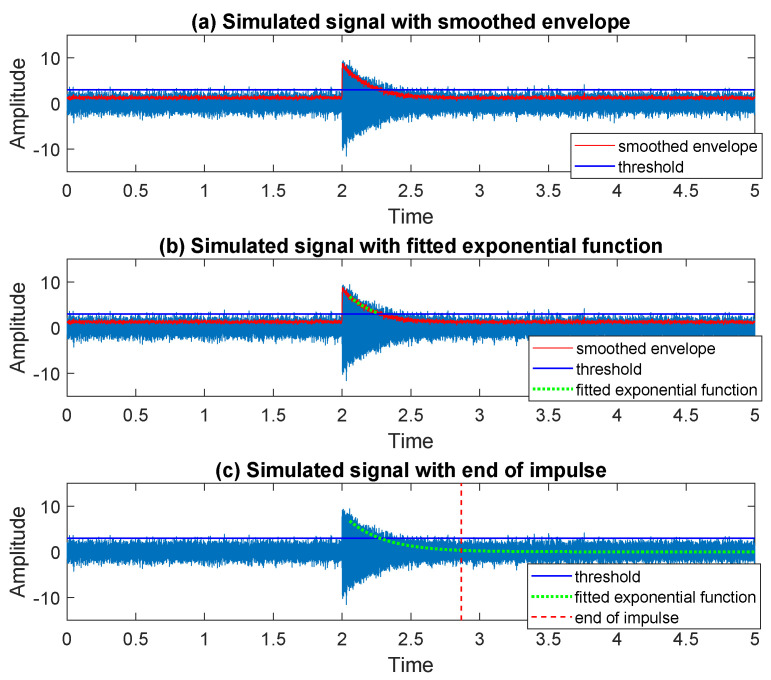
The consecutive steps of the procedure leading to the end recognition for an impulse in the simulated signal.

To identify the moment when the impulse starts, on the Figure 7a we plot a part of the signal around the sample determined by the moving maximum in the previous step. Following the procedure proposed in Section 3.3, we calculate the moving range (in Figure 7b) together with the corresponding empirical probability density function (in Figure 7c). As one can see, the distribution of the values taken by the moving range is bi-modal. By finding the local minimum separating the modes, we divide the values taken by statistic into two groups related to the area before impulse and the area of impulse, see in Figure 7d where the start of the impulse is marked with a red dashed line. In the next step, to find the end of the detected impulse we apply the method described in Section 3.4. The consecutive steps of the procedure are presented in the Figure 8a–c. The threshold chosen to determine the end of the approximation interval, for the exponential function fitted to the envelope of the signal, is equal to 3 (three times the standard deviation of noise—according to the 68–95–99.7 rule). The end of the impulse is marked with a red dashed line in Figure 8c.

**Figure 9:**
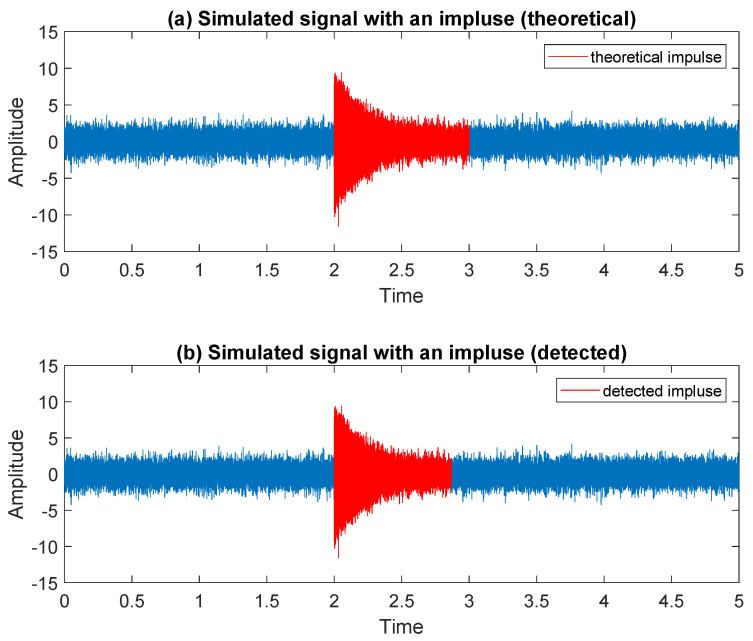
Simulated signal with an impulse marked in red: theoretical—panel (**a**), and detected using the proposed methodology—panel (**b**).

To summarize the results obtained in this section, in Figure 9 we present the simulated signal with the impulse marked in the original location—panel (a), and with the impulse marked in the location determined using the proposed methodology—panel (b).

### 4.3. Simulation Study on the Influence of Impulse Amplitude on the Procedure Efficiency

**Figure 10:**
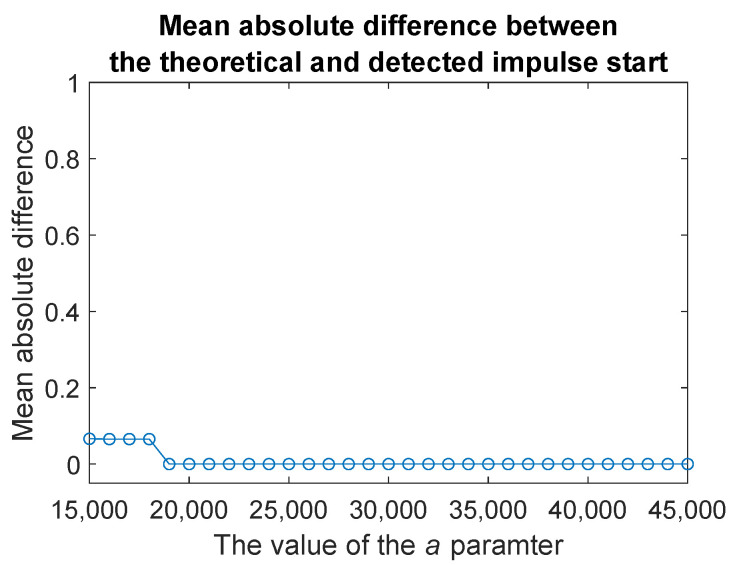
Mean absolute difference between the theoretical and detected impulse start calculated based on M=100 simulated signals with exponentially decaying impulse as in Equation (7) where b=4, f=3000 and *a* is changing from 15,000 to 45,000.

**Figure 11:**
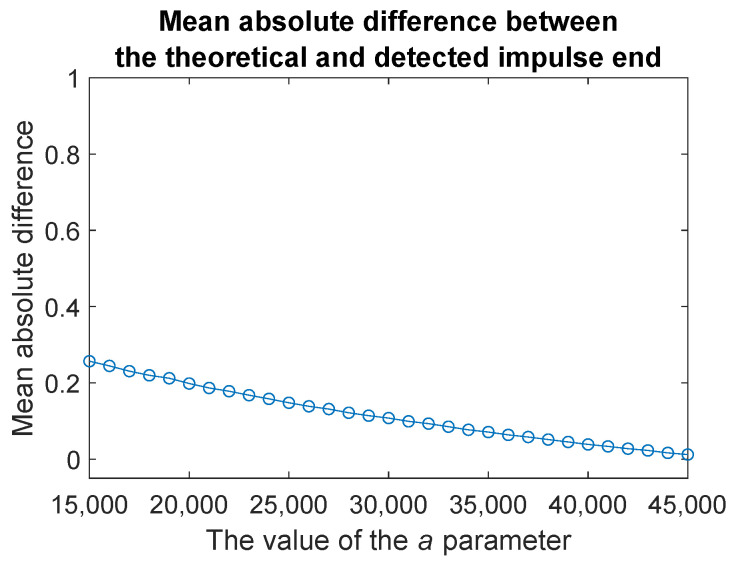
Mean absolute difference between the theoretical and detected impulse end calculated based on M=100 simulated signals with exponentially decaying impulse as in Equation (7) where b=4, f=3000 and *a* is changing from 15,000 to 45,000.

To evaluate the influence of the parameter *a* in Equation (7), which is responsible for the amplitude of the generated impulse, on the correctness of the proposed procedure, we conduct additional simulation study. For each *a* changing from *a* = 15,000 to *a* = 45,000 with step equal to 1000, we generate M=100 simulated signals as described above. For each signal, we localize the impulse using the method proposed in the paper. Then, we determine the absolute value of the differences between the theoretical values of its beginning and its end and those values that are determined by using our method. The results are presented in Figure 10 and Figure 11.

In both figures, on the *y*-axis we plot the mean absolute differences between the theoretical and determined values. We can notice that in both cases, the method works better as the parameter *a* increases. The clearer the impulse, the easier it is to correctly determine its location. Moreover, the procedure for detecting the start of an impulse relatively quickly becomes error-free looking at the mean of the absolute difference.

## 5. Real Data Analysis

In this section, we analyze the real acoustic signal described in Section 2. To locate all impulses in the data, we proceed according to the presented methodology, and the following subsections correspond to the subsequent stages of the proposed procedure.

### 5.1. Iterative Removal of Deterministic Components

Following the methodology introduced in Section 3.1, to remove the deterministic components, we divide the signal into several segments. For each segment, we fit a sine function to the data. Then, we examine the frequencies corresponding to the subsequent sine functions (the number of sine functions is equal to the number of segments). In Table 2 for all considered segment lengths we present the sample variance of these frequencies. It can be seen that for the segments of length 40,000, the calculated sample variance takes the smallest value. For such a length, the frequencies of the fitted sines take very similar values in all segments.

**Figure 12:**
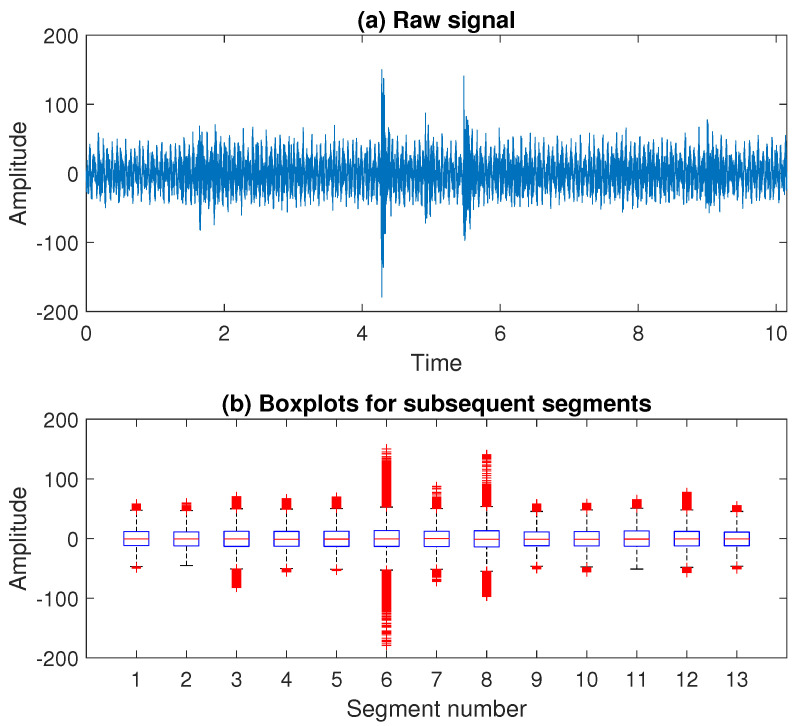
The raw signal and the boxplots presenting the values in the subsequent segments.

**Figure 13:**
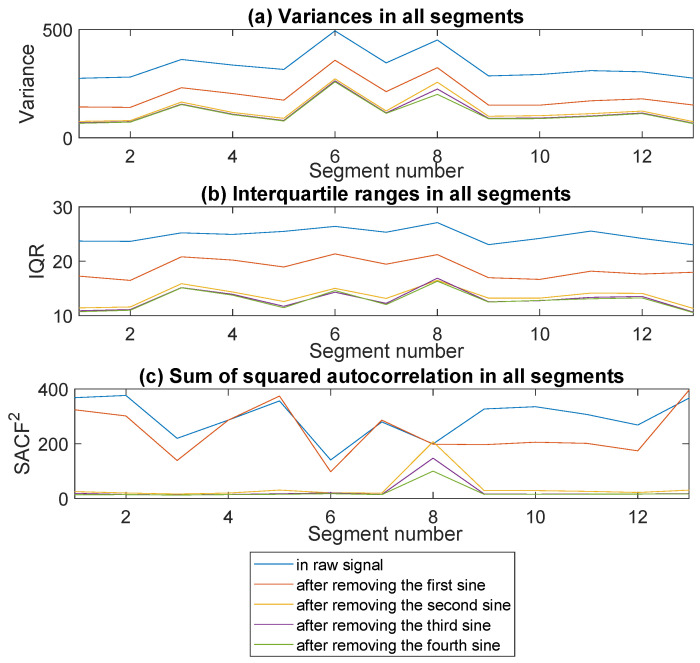
Sample variance, interquartile range and sum of squared auto-correlation calculated for the subsequent segments for the raw signal and the signals obtained after removing the sine functions.

**Figure 14:**
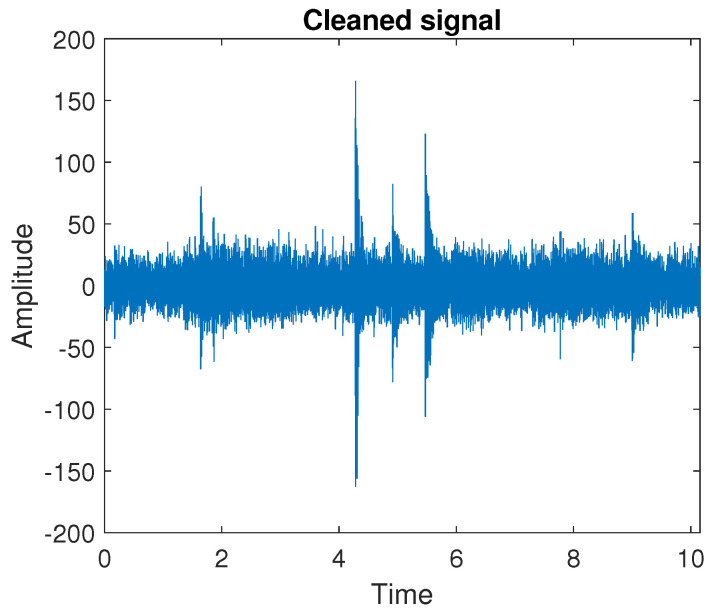
The signal obtained after removing the deterministic part.

Therefore, we divide the signal into 13 segments with 40,000 samples in each of them. The raw signal and the boxplots presenting the values in subsequent segments are presented in Figure 12. In the next step, we fit the sine function to each segment separately and compare the statistics determined for residual time series obtained after removing the matched functions. In Figure 13, one can see how the sample variance, IQR, and ${\mathrm{SACF}}^{2}$ calculated for all segments change after subtracting consecutively first, second, third, and fourth sine function compared to the raw signal. It can be seen that all the statistics take much lower values, compared to the raw data, after removing the first and the second sine. Further operations do not make such a significant difference, therefore we decide to consider the signal cleaned by subtracting consecutively two sine functions. The signal obtained after removing the deterministic part is presented in Figure 14. One can notice that for the cleaned signal, the amplitude decreases and the impulses present in the data are more visible.

### 5.2. Detecting the Number of Impulses

**Figure 15:**
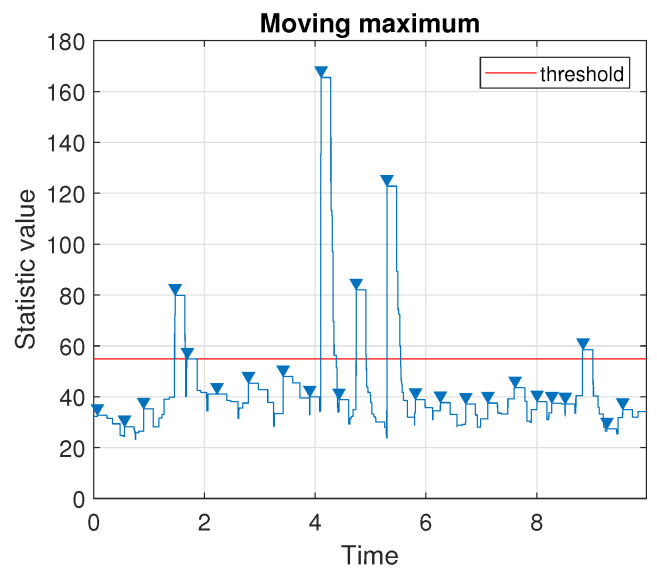
Values taken by the moving maximum calculated over a sliding window of length 9000. Triangle markers indicate the local peaks and the threshold is chosen as a quantile of order 0.875.

Using the method presented in Section 3.2 we determine the number of impulses in the signal based on the values taken by the moving maximum, see Figure 15. As the threshold indicating the presence of an impulse, we choose the quantile of order 0.875 of the calculated statistics. One can observe that the method leads to detecting six impulses in the analyzed signal. In the following two subsections, we describe in detail how to use the proposed methodology to determine the start and the end for one of the impulses visible in the data. The signal with all six detected impulses marked in their locations will be presented at the end of the section.

### 5.3. Impulse Start Recognition for an Exemplary Impulse

**Figure 16:**
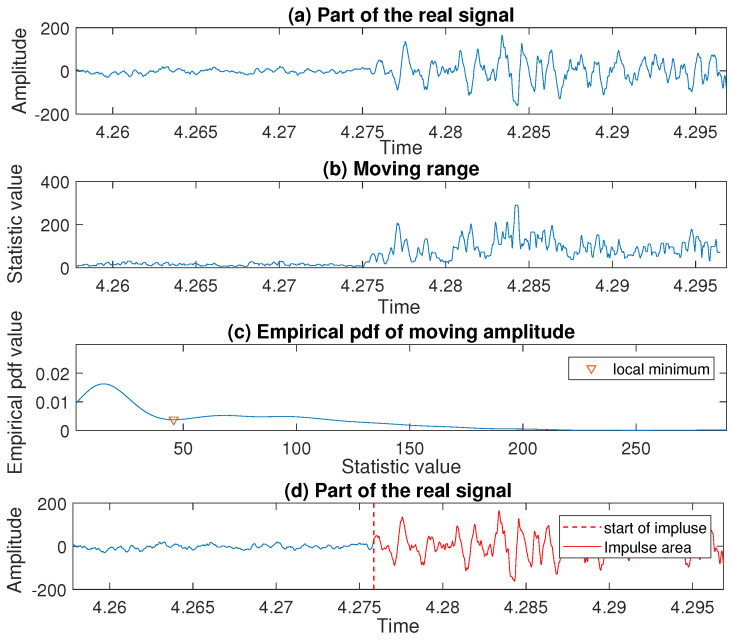
The consecutive steps of the procedure leading to the start recognition for an exemplary impulse in the real signal.

Let us consider the third impulse detected in the signal on the previous stage of the analysis, namely, the one with the peak located between t=4 and t=4.5. To find its start we follow the procedure presented in Section 3.3. The consecutive steps are shown in Figure 16, where we present a part of the real signal—panel (a), the values taken by the moving range statistic—panel (b), and the corresponding empirical probability density function—panel (c). Finally, on panel (d) one can see the moment when the impulse starts marked with a red dashed line. As mentioned above, the same procedure is used to detect the beginning of all five remaining impulses present in the signal.

### 5.4. Impulse End Recognition for an Exemplary Impulse

**Figure 17:**
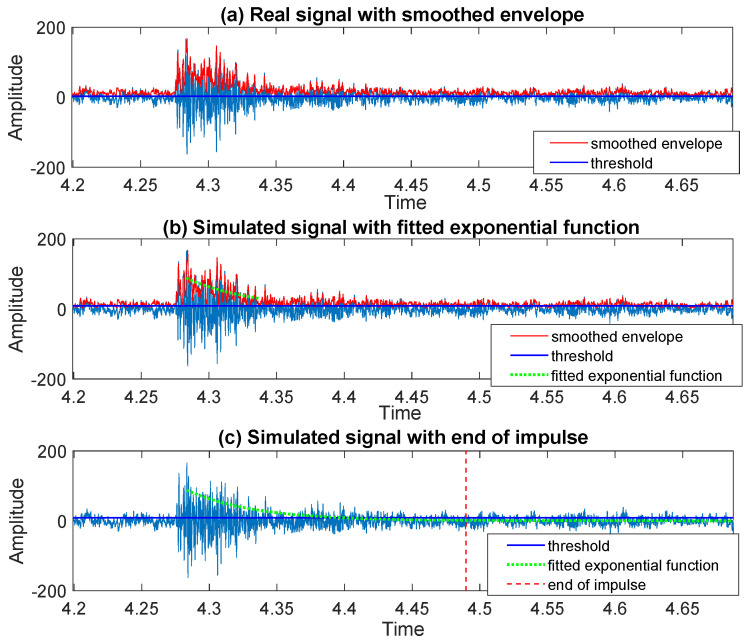
The consecutive steps of the procedure leading to the end recognition for an exemplary impulse in the real signal.

**Figure 18:**
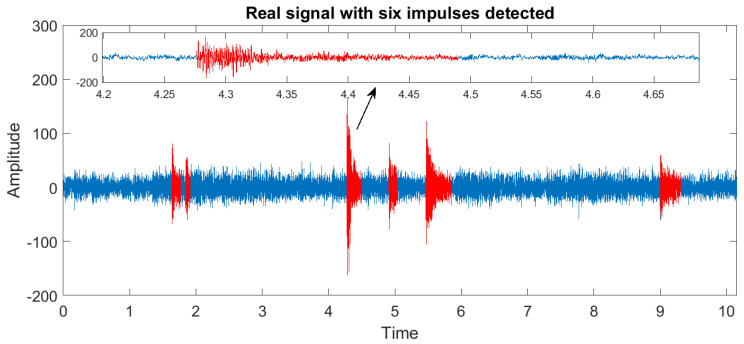
The location of all six impulses in the real signal.

For the impulse starting at the moment marked in Figure 16, we detect the end by using the methodology proposed in Section 3.4. All required steps are presented in Figure 17. Panel (a) shows the calculated smoothed envelope of the signal and the threshold chosen as the standard variation of part of the data where there are no impulses present (that is, the first 1.5 s of the signal). On panels (b) and (c), one can see the exponential function fitted to the signal envelope and its extension together with the end of the impulse marked with a red dashed line, respectively. The same methodology is applied to identify the end of all five remaining impulses present in the signal. The exact location of the third impulse considered here, taking into account its end and its beginning identified in the previous section, can be seen on the zoomed panel in Figure 18, which also shows the other five impulses detected in the signal.

## 6. Summary and Discussion

In this paper, a novel procedure for the identification, decomposition, and segmentation of impulsive vibration signals with complicated, time-varying structure has been proposed. The developed algorithm consists of a few steps that allow for effective deterministic component removal and impulses recognition.

The proposed methodology first is based on the identification and removal of several deterministic components (the sine wave functions). Due to AM/FM modulation (the amplitudes and frequencies of the sine wave function change over time), this step is based on segmentation and the identification of the deterministic components separately for each segment of the signal. That is a crucial step in the further analysis. Segment size, as well as the number of iterations, is derived experimentally, statistical-based criteria have been proposed here.

This step could be sensitive to large AM/FM modulations, which may happen for seriously varying speed (machine start/stop procedures). For such a very non-stationary regime, the size of the segment will be very short and it may result in poor approximation (fitting). However, it should be noted that from practice we know that the machine is in operation continuously, so the number of start/stop procedures is not significant.

After the deterministic component removal, we have proposed a new algorithm of impulse identification. Separately we detect the beginning of the impulse and its end as well as the peak amplitude of each impulse. The efficiency of this step will decrease for impulses with smaller amplitudes. In this algorithm, there is a need for several samples that protrude from the signal to build a model of envelope (to fit the exponential function). It is obvious that for weak impulses, even after deterministic component removal, several samples will be smaller so the precision of fitting will be worse. However, it should be noted that in the real environment we consider the signals with large amplitudes impulses (even 20–30 times larger than the averaged level of the noise). For small amplitudes impulses, there is no reason to use our procedure.

The proposed algorithm, which consists of a few important steps, is used for the very complicated signal coming from the sieving screen. Our idea was to propose a relatively intuitive method that can be used in real applications. Here we proposed a step-by-step procedure which effectiveness is demonstrated for the simulated signals. Finally, we applied this methodology to the signal from the machine working in a real environment.

Although the proposed algorithm seems to be sufficient in the considered case, there are additional limitations. The procedure is dedicated to the signals related to the machines with quasi-stationary work. In another case, it will be difficult to identify the optimal segments’ length in the first step of the procedure and to fit the optimal sine wave functions. The second case that may cause the proposed method to be ineffective, is related to the impulses overlapping. When one impulse starts before the end of the previous one, the algorithm can return the wrong structure break point (i.e., the beginning of the new impulse and its end). Last but not least is the issue related to the number of impulses. It is obvious that a large number of impulses will extend the operation time of the entire procedure.

## Figures and Tables

**Table 1:** The values of variance, interquartile range, and ${\mathrm{SACF}}^{2}$ calculated for the signal with deterministic components and for the residual signals obtained after subtracting the first, second, third, and fourth sine function (denoted as $r_{1}$, $r_{2}$, $r_{3}$ and $r_{4}$, respectively).

	Signal with Deterministic Components	$r_{1}$	$r_{2}$	$r_{3}$	$r_{4}$
variance	3.59	2.61	1.88	1.79	1.68
IQR	2.44	1.97	1.45	1.49	1.49
${\mathrm{SACF}}^{2}$	1003.66	723.86	140.07	131.44	103.60

**Table 2:** The length of one segment (the number of samples in one segment) and the corresponding sample variance calculated for the frequencies of the sine functions fitted to all segments.

**Length**	10,000	15,000	20,000	25,000	30,000	35,000	40,000	45,000	50,000	55,000
**Variance**	5505.8	8341.7	0.0117	14213	6.4279	23.5842	0.0003	15.236	10.336	23.805
